# Influence of the Psychological Effect of COVID-19 on Saudi Student Nurses’ Attitudes toward Nursing and Their Future Career Plans

**DOI:** 10.3390/jpm13040672

**Published:** 2023-04-17

**Authors:** Khalaf Aied Alotaibi, Ejercito Mangawa Balay-odao, Jonas Preposi Cruz, Jazi Shaydied Alotaibi, Junel Bryan Bajet, Nahed Alquwez, Jennifer Mesde, Ahmed Mansour Almansour, Ahmed Almoghairi, Abdulrhman Saad Albougami

**Affiliations:** 1 Department of Nursing, College of Applied Medical Sciences, Shaqra University, Dawadmi 11961, Saudi Arabia; 2 Department of Medicine, School of Medicine, Nazarbayev University, Kerey and Zhanibek Khans St 5/1, Nur-Sultan 010000, Kazakhstan; 3 School of Advanced Studies, Saint Louis University, Baguio City 2600, Philippines; 4 Department of Nursing, College of Applied Medical Sciences, Majmaah University, Al-Majmaah 11952, Saudi Arabia

**Keywords:** attitudes, career plan, COVID-19, nursing education, psychological effect, Saudi Arabia

## Abstract

Background: The effects of COVID-19 on the attitude of student nurses toward the nursing profession remain unclear because few studies have been conducted on this topic. Thus, this study examines the influence of the psychological effects of COVID-19 on student nurses’ attitudes toward the nursing profession and their desire to become nurses. Method: The study utilised a quantitative, cross-sectional, and observational design. It surveyed a convenience sample of 726 student nurses in Saudi Arabia during the first semester of the 2021–2022 academic year. Results: The students reported low levels of COVID-19 fear, anxiety, stress, phobia, and obsession. The students reported positive attitudes toward the nursing profession and 86.0% highlighted their desire to continue with nursing as their future profession. Gender, knowing someone infected with COVID-19, confidence in the government’s pandemic response, fear, anxiety, and phobia were significant predictors of the nurses’ attitudes. Community, family members in the profession, “COVID-19-related anxiety”, and “preference for the nursing profession” were significant predictors of the student’s desire to continue nursing. Conclusions: Living in a rural community, having family members in the nursing profession, having low levels of COVID-19-related anxiety, and having positive attitudes toward nursing increased the likelihood of nursing students continuing their nursing careers during the COVID-19 pandemic.

## 1. Introduction

Nursing is a profession that ensures the provision of holistic care to clients throughout their lives. It is considered to be a humanitarian service because nurses provide care to people regardless of their socioeconomic status, gender, race, and age. Recently, nurses have played a pivotal role in preventing, treating, and controlling COVID-19. As nurses have delivered healthcare during the COVID-19 pandemic, unintended problems, such as physical fatigue, psychological issues, and negative attitudes from the community, have become inevitable [1]. In some instances, nurses are shunned and physically and verbally abused by the community because they are perceived as spreaders of the virus [2]. These nurses’ experiences influenced the researchers’ curiosity in determining the psychological impacts of COVID-19 on student nurses and how it has impacted their attitudes toward the nursing profession.

An infectious disease outbreak causes psychological distress throughout the whole community both during its occurrence and after it ends [3]. In a study conducted in Israel, over 244 nursing students showed increased rates of anxiety and fear of infection [4]. Student nurses in three different universities in the eastern region of Turkey were surveyed and their stress levels were assessed. The students reported moderate stress levels due to the COVID-19 pandemic [5].

The COVID-19 pandemic has brought recognition to the nursing profession and nurses have been praised for their heroic deeds. Society’s positive feeling toward nurses has uplifted their morale and dignity and motivated them to remain committed to their oath to provide patient care even if their health is at risk. However, nurses have also experienced discrimination and have been shunned for being carriers and spreaders of COVID-19 [1]. The stigma associated with infection has affected nurses’ mental health and nurses have experienced fear and anxiety about the possibility of infecting their family members with the virus [6]. Furthermore, nurses have often been infected with COVID-19, which could threaten nursing students with exposure to infectious diseases [7]. The fear of COVID-19 infection during clinical exposure is a central concern of nursing students [8]. Therefore, some nursing students have left their programs due to the impact of COVID-19 [9]. However, despite COVID-19, some student nurses have decided to continue nursing and have recognised the important role of the nursing profession in the community [8,9]. Furthermore, Koltai et al. [10] mentioned that vaccinating against COVID-19 positively impacted people’s mental well-being and lowered the stress levels associated with contracting the virus.

The impact of COVID-19 on student nurses’ mental health and attitude toward the nursing profession can be a notable barrier to the pursuit of nursing. Evidence shows that students have been psychologically and mentally affected by the COVID-19 pandemic [11]. It has undoubtedly caused stress for students due to fear of catching the virus. This affects nursing students’ mental or psychological health, reducing their interest and motivation to study [12]. Therefore, examining how the COVID-19 pandemic has affected students’ attitudes toward the nursing profession is essential. However, the psychological effects of COVID-19 on the attitude of student nurses toward the nursing profession remain unclear because only a few studies have been conducted on this topic. Furthermore, the limited literature on this topic does not illuminate the impacts of COVID-19 on student nurses. Insight into this topic would considerably improve knowledge of the needs, issues, concerns, and difficulties of nursing students in Saudi Arabia. Therefore, this study addresses this gap by aiming to understand the influence of the psychological impacts of COVID-19 on Saudi student nurses’ attitudes toward the nursing profession and their desire to become a nurse.

### Aims

This research examined the influence of the psychological effects of COVID-19 on student nurses’ attitudes toward the nursing profession and their desire to become nurses. It also examined the other factors (i.e., demographics and COVID-19-related variables) that influence student nurses’ attitudes toward the nursing profession and their career intentions.

## 2. Materials and Methods

### 2.1. Design

This survey utilised a quantitative research design. Specifically, it used cross-sectional and observational designs.

### 2.2. Setting and Samples

This study was conducted on Saudi student nurses studying at government universities in Saudi Arabia. While the study was being conducted, many COVID-19 cases were still reported in the country. However, the government had already implemented measures for prevention of transmission and mitigation of the impacts of the pandemic. The government had implemented a vaccination program nationwide. The vaccination rate during the time was high. At the height of the pandemic, all universities were closed, and education was delivered online. However, during the data collection period, universities started to open and some classes (i.e., major nursing courses) were brought back to being in person. Clinical duties of students in some universities resumed, with limitations, but clinical duties in some universities were still suspended. Clinical practice was replaced by nursing laboratory activities. In total, 726 student nurses were surveyed electronically using a convenience sampling technique. The participants fulfilled the following inclusion criteria: (1) Saudi nationals, (2) enrolled in a nursing program proper or a preparatory year level for medical-related programs at any government university in the country, (3) have access to the internet, and (4) are willing to participate. The exclusion criteria for this study included (1) non-Saudis, (2) belonging to other programs, and (3) inability to access the Internet.

### 2.3. Ethical Considerations

An Ethical Review Committee approved this research. This research followed the highest ethical standards for survey studies. The students were fully informed and given disclosure about the study’s information. These details were given in the first part of the online survey. The students who agreed to participate signed an electronic informed consent form. Once the students input their initials to signify their voluntary participation, they were directed to click “Next” to resume the online survey. By not collecting this information, the participants’ identity was protected.

### 2.4. Instrument

An online survey containing the following sections was used to collect the data to meet the study’s objectives:

The participants’ age, sex, year level, community type, and family type were queried. The question “Do you have a family member/s working as a nurse/s?” was also asked.

The following questions were asked: (1) “Have you tested positive for COVID-19?”; (2) “Did anyone from your family become infected with COVID-19?”; (3) “Do you know someone in your immediate social environment who has been infected with COVID-19?”; (4) “How serious is the COVID-19 pandemic in your community?”; (5) “How serious is the COVID-19 pandemic in Saudi Arabia?”; and (6) “How confident if at all, are you that the government is doing a good job responding to the COVID-19 pandemic?”.

The students used the “Fear of COVID-19 Scale” (FCV-19S) to assess their “fear of COVID-19”. This unidimensional scale had a five-point response option that ranged from “strongly disagree” (1) to “strongly agree” (5). The scores were calculated by obtaining the sum of the tool’s seven items, which could range from 7 to 35. High scores were interpreted as high levels of COVID-19-related fear. This tool had excellent reliability, as evidenced by its internal consistency (α = 0.82) and stability reliability (ICC = 0.72). The correlation of the FCV-19S with the “Hospital Anxiety and Depression Scale” and the “Perceived Vulnerability to Disease Scale” supported its concurrent validity [13].

The “Coronavirus Anxiety Scale” (CAS) was used to measure the participants’ COVID-19-related anxiety. The CAS asked the participants to answer questions by choosing options that ranged from “not at all” (0) to “nearly every day” (4). The options reflected anxiety symptoms, such as “dizziness, sleep disturbances, tonic immobility, appetite loss, and abdominal distress”. The scores were calculated by summing the item scores (range = 0 to 20). The α of the scale was 0.93 [14].

The “Depression, Anxiety and Stress Scale short form version” (DASS-21) [15] was used to assess stress. This 21-item tool (0 = never to 3 = almost always) was used to measure the severity of the “core symptoms of depression, anxiety, and stress” reported by the participant. The DASS-21 allows the measurement of the severity of an individual’s symptoms; however, it cannot be used to categorically assess for clinical diagnosis. In this study, only the stress subscale was used. The stress score was calculated by summing the scores of the items belonging to the stress subscale and multiplying the total by two. A score of 0 to 14 was “normal”, 15 to 18 was “mild”, 19 to 25 was “moderate”, 26 to 33 was “severe”, and 34 and above was “extremely severe”. The internal consistency of the stress subscale of DASS-21 ranged from 0.81 to 0.95 across adult and adolescent samples. The three subscales under DASS-21 had high correlations [16,17].

COVID-19-related phobia was assessed using the “COVID-19 Phobia Scale” (C19P-S) [17]. C19P-S’s items address the specific diagnostic criteria used for phobia based on DSM-V. This scale had 20 items with five-point response choices that ranged from “strongly disagree (1)” to “strongly agree (5)”. C19P-S had four subscales: “psychological factors, psychosomatic factors, economic factors, and social factors”. A subscale score and an overall score were calculated from this tool. C19P-S’s reliability was high (α = 0.925), and its construct validity was supported by EFA and CFA. Moreover, C19P-S has been shown to have excellent convergent and discriminant validities [18].

Obsession with COVID-19 was measured using the “Obsession with COVID-19 Scale” (OCS) [18]. The OCS is a self-reported tool that can be used to screen mental health, specifically, persistent and disturbing thoughts regarding COVID-19. The scale was created to effectively determine cases of functional impairment associated with a person’s thinking patterns related to COVID-19. The scale comprised four items with response choices ranging from 0 (“not at all”) to 4 (“nearly every day”). Participants’ scores for each item were added and scores ≥ 7 were interpreted as “probable dysfunctional thinking about COVID-19”. The tool has been proven to be reliable (α = 0.83) and valid (construct validity) [19].

Participants’ attitude toward the nursing profession was measured using the “Attitude Scale for Nursing Profession” (ASNP) [20]. The ASNP consisted of 40 items, which were grouped into three categories: “properties of the nursing profession”, “preference for the nursing profession”, and “general position of the nursing profession”. Participants answered all questions using a five-point Likert scale (1 “I strongly disagree” to 5 “I completely agree”). As 8 of the 40 items were worded negatively, their scores were reverse coded before inclusion in the analysis. The overall mean score and subscale mean scores were calculated. A mean score of over three indicated a positive attitude, whereas a mean score below three indicated a poor attitude. For this study, only the subscales “preference for the nursing profession” and “general position of the nursing profession” were used because these constructs were the focus of the research. The computed Cronbach’s α for ASNP was 0.91. EFA and CFA confirmed the three-factor solution of the scale, indicating its construct validity [20].

Willingness to continue with nursing as a career was measured using the following “yes–no–not sure” question: “Based on your experience during the COVID-19 pandemic, do you still want to pursue nursing as your future career?”.

### 2.5. Data Collection

The online survey was commissioned during the first semester of the 2021–2022 academic year. Recruitment of participants were conducted online by the researchers. The invitation to participate (i.e., recruitment message) in the survey was forwarded to potential participants using social media ((i.e., WhatsApp, Facebook, Twitter, Instagram) and email. The invitation included the study information and the link to the online survey. Additionally, the recruitment message indicated that only those nursing students from government universities were included in the study. Those willing to join were instructed to click on a link that directed them to the online informed consent form. The participants who clicked “I agree to participate” were directed to the first part of the online survey. By contrast, those who clicked “I do not agree” were automatically exited from the survey. The online survey was open until the end of the data collection period. Data were extracted from the online survey and analysed accordingly.

### 2.6. Analysis of Data

Descriptive analyses were used for demographic and COVID-19-related information. Likewise, descriptive analyses were carried out for the other variables, namely, COVID-19-related fear, stress, phobia, obsession, attitudes toward the nursing profession, and willingness to continue with nursing. Standard multiple regression analysis was used to determine the factors associated with attitudes toward the nursing profession. Binary logistic regression was carried out to identify predictors of students’ willingness to continue with nursing. Statistical significance was determined if the *p*-value was less than 0.05.

## 3. Results

Table 1 summarises the participants’ demographics and COVID-19-related perceptions and experiences. The mean age of the participants was 23.24 (SD = 4.83) years. Most of the students were females (67.6%), living in a rural community (54.3%), with nuclear families (60.2%), and with family members who are in the nursing profession (55.0%). The number of participants in year two of the BSN program was the highest (34.8%), followed by year four students (22.6%), year three students (18.7%), internship-year students (15.6%), and preparatory-year students (8.3%). Less than half of the students had previously been infected with COVID-19 (46.7%), whereas more than half of the students had family members (72.0%) and had known someone in their immediate social environment (75.2%) who had been infected with the virus. On a scale of 0 to 100, the mean score of the students’ consideration of the seriousness of the pandemic in Saudi Arabia was 47.73 (SD = 27.54). Most of the students were very confident that the government’s pandemic response had been effective (77.4%).

### 3.1. Descriptive Results of the Study Variables

The students reported low levels of COVID-19-related fear (M = 12.78, SD = 5.90), anxiety (M = 1.17, 2.61), stress (M = 7.60, SD = 8.22), phobia (M= 38.12, SD = 16.64), and obsession (M = 2.48, SD = 3.08) (see Table 2). A total of 81.8% (*n* = 594) of the participants were categorised as having normal stress levels, while 7.4% (*n* = 54), 6.9% (*n* = 50), 2.9% (*n* = 21), and 1.0% (*n* = 7) were categorised as having mild, moderate, severe, and extremely severe stress, respectively. For “obsession”, 91 student nurses (12.5%) had scores that qualified for the cut-off (≥7) for “probable dysfunctional thinking about COVID-19”. The remaining participants recorded mean scores < 7.

As indicated in Table 2, the participants’ average score for the “preference for the nursing profession” dimension of the variable “attitudes toward the nursing profession” was 4.16 (SD = 0.74). By contrast, the mean score for “general position of the nursing profession” was 3.77 (SD = 0.46). Furthermore, most of the participants (86.0%) highlighted their desire to continue with nursing as their future profession based on their experiences during the pandemic. However, 8.5% (*n* = 62) reported they were not sure if they still wanted to continue with nursing. Approximately 5.5% (*n* = 40) of the students mentioned that they did not wish to continue nursing following their experiences during the pandemic.

### 3.2. Predictors of Attitudes toward the Nursing Profession

The multiple regression analysis shows the variables that significantly predicted the student nurses’ attitude toward the nursing profession (*F_16,70_*_9_ = 8.78, *p* < 0.001). The regression model was significant and explained 14.7% (*R*^2^ = 0.165) of the variance in students’ attitudes toward the nursing profession. As shown in Table 3, gender, knowing someone infected with COVID-19, confidence in the pandemic response of the government, fear, anxiety, and phobia were significant predictors of the nurses’ attitudes. Specifically, being female was associated with having positive attitudes (ß = 0.13, *p* = 0.001, 95%CI = −0.02, 0.00). Knowing someone in their immediate social environment who was infected with COVID-19 was also associated with positive attitudes (ß = 0.13, *p* = 0.007, 95%CI = 0.04, 0.22). Having confidence in the government’s response to the pandemic was related to positive attitudes (ß = 0.13, *p* < 0.001, 95%CI = 0.06, 0.19). Having significant COVID-19-related fear (ß = −0.03, *p* < 0.001, 95%CI = −0.04, −0.02) and anxiety (ß = −0.03, *p* = 0.006, 95%CI = −0.05, −0.01) was associated with poor attitudes toward the nursing profession. However, having COVID-19-related phobia was linked to positive attitudes toward the nursing profession (ß = 0.01, *p* = 0.004, 95%CI = 0.00, 0.01).

### 3.3. Predictors of the Students’ Willingness to Continue with Nursing as a Future Profession

The results of the binary logistic regression are summarised in Table 4. The model was statistically significant (χ2[18] = 197.89, *p* < 0.001) and explained 42.9% (Nagelkerke *R^2^*) of the variance in the students’ willingness to pursue nursing as a profession. The model correctly classified 88.7% of the cases. The regression identified community, family members in the profession, “COVID-19-related anxiety”, and “preference for the nursing profession” as significant predictors of the students’ willingness to continue with nursing. Specifically, student nurses living in rural communities were 1.83 times more likely to wish to continue nursing than those living in urban areas (Exp[ß] = 1.83, *p* =.031, 95%CI = 1.06, 3.17). The probability of students living in rural areas continuing with nursing was 64.7%. Students with family members in the nursing profession were 2.21 times more likely to continue their nursing careers than those without family members who were nurses (Exp[ß] = 2.21, *p* = 0.007, 95%CI = 1.25, 3.92). The probability of the students who had nurses as family members continuing with nursing was 68.8%. Moreover, every unit increase in the stress score was associated with a 4% decrease in the likelihood of continuing with nursing (Exp[ß] = 0.96, *p* = 0.031, 95%CI = 0.93, 1.00). Every unit increment in the “preference for the nursing profession” score was associated with a 733% increase in the likelihood of continuing with nursing (Exp[ß] = 8.33, *p* < 0.001, 95%CI = 5.22, 13.29).

## 4. Discussion

This research has examined the influence of the psychological effects of COVID-19 on student nurses’ attitudes toward the nursing profession and their desire to become nurses. COVID-19 caused high levels of stress and anxiety among nursing students [4]. In the investigation by Tekir [21], the level of COVID-19 fear was below moderate. The results of other investigations have indicated that student nurses experienced moderate stress and anxiety levels [5]. However, this study showed that student nurses had low levels of “fear, anxiety, stress, phobia, and obsession” concerning the COVID-19 pandemic. This finding confirms the results reported by Alsolais et al. [11], who found that student nurses in Saudi Arabia experienced a low degree of “depression, anxiety, and stress” during the pandemic. This may be attributed to the time the study was conducted, during which most people had become used to practising the health protocols implemented by the Ministry of Health of Saudi Arabia. Furthermore, most of the students had received their second and booster doses of the COVID-19 vaccine; Koltai et al. [10] found that having the COVID-19 vaccine improved a person’s psychological well-being. Therefore, it is possible that the student nurses’ generally good mental health status could be attributed to the COVID-19 vaccine. This notion could be related to the desirable coping behaviours of the nursing student, which is reality-centred [22], since they decided to take realistic and practical measures, such as having the COVID-19 vaccine, to prevent the spread and being infected with the disease.

This study further showed that the student nurses had positive attitudes toward nursing. This contradicts the results of Cici and Yilmazel [23], who reported negative views of the nursing profession during the pandemic. Additionally, previous studies have found that nursing students intended to leave nursing school due to the pandemic [9]. However, other studies have revealed that student nurses’ attitudes toward the nursing profession were positive before and during the pandemic [24]. This indicates that despite the adverse effects of the pandemic, the student nurses retained their positive outlook on their profession. Other studies have even reported that student nurses felt enthusiastic about caring for COVID-19 patients [25].

The positive attitude of the student nurses may be associated with the appreciation that nurses received during the pandemic. Throughout the media, nurses were applauded for their unconditional responsibilities and duties in providing nursing care to COVID-19 patients. Notably, nursing welfare was the government’s priority during the pandemic, and Saudi Arabia had well-established healthcare policies and guidelines implemented to protect nurses. These factors may have created a positive image of the nursing profession for student nurses and motivated them to continue nursing as their future profession. Therefore, despite the pandemic’s negative effects, nursing students are willing to pursue nursing due to their desire to provide care regardless of their patient’s condition and even if a patient is infected with COVID-19 [26]. Student nurses with positive attitudes toward their profession believed that the pandemic influenced their desire to continue and helped them to reinvent their professional identities [24].

Being female was linked to having a highly positive attitude; this finding is consistent with the results for most countries [27]. A previous study indicated that female nurses were enthusiastic about providing care to COVID-19 patients [28]. In other situations, female nursing students were reported to have more positive attitudes than male nursing students [29]. This was reflected during the COVID-19 pandemic [24]. Furthermore, women dominate the nursing profession and are more likely to be healthcare providers than men, which may have influenced the above result.

The nursing students’ positive attitude toward the nursing profession was associated with having COVID-19-infected family members. Shah et al. [30] stated that the psychological effects of seeing a person infected with COVID-19 motivated nursing students to provide nursing care. Moreover, the idea that their family members could be infected with COVID-19 influenced the willingness of nursing students to continue nursing [31]. This finding highlighted the potential positive influence of the nursing students’ moral obligation or duty to provide care to others on their attitudes toward nursing [32], particularly if their family members are at risk or involved.

In addition, the respondents’ confidence in the government’s pandemic response was associated with their positive attitudes. The Saudi government’s actions to control the COVID-19 pandemic significantly contributed to this finding. The response of the Saudi government reasonably decreased the number of cases and mortalities associated with COVID-19 [33]. The nursing students felt confident in the Saudi government and the Ministry of Health’s response to promote the nation’s awareness and involvement in controlling the pandemic [34]. The students became highly resilient due to the government’s pandemic response efforts.

Regression analysis revealed that four key factors contributed to the respondents’ willingness to continue their nursing careers. First, students living in rural areas had the greatest desire to continue nursing. This finding could be associated with the increasing employment opportunities for nurses in rural areas in the country. Compared with other students, students living in urban communities have more employment options and broader social connections and networks, which may affect their career choices. This idea is supported by Chuenkongkaew et al. [35], who reported that students who studied in rural areas were likely to stay and work in those places. However, this study’s findings contradict the results of Bahçecioğlu Turan et al.’s [24] study, which reported that nursing students who studied in urban areas where viral infections are common were more likely to complete the program than those who studied elsewhere.

Having family members who are nurses also influenced the respondents’ willingness to continue with the nursing profession. The findings showed that having a family member in the nursing profession influenced the nursing students’ positive attitudes toward the nursing profession because they motivated them to pursue a career in nursing. Kandil et al. [36] identified that having a parent who is a nurse influenced the preference of nursing students toward nursing education. Increased stress decreased the likelihood of students continuing with nursing. This finding could be associated with the statements of Labrague and Santos [37] and Nashwan et al. [38]. As increased anxiety motivated the turnover intention and psychological distress of front-line workers, the increased anxiety due to COVID-19 experienced by students influenced their view of the nursing profession and their future career. This finding could also be linked to the nurses’ stories of negative experiences during the pandemic, such as psychological distress, uncertainties, and an unsafe workplace [39], which may have affected their desire to continue nursing.

Lastly, nursing students who preferred nursing as their profession had increased odds of continuing with nursing. Specifically, the subscale “preference for the nursing profession” was a significant predictor of the students’ desire to continue with nursing. The increased appeal of the nursing profession to nursing students who prefer the nursing profession could be associated with societal recognition of the nursing profession during the pandemic [8]. This positive feedback from society to the nursing profession uplifted the morale and dignity of nurses. The desire of nursing students to continue nursing increased because they felt that they have an important role to play in public health crises, such as the COVID-19 pandemic.

### Limitations

First, sample selection was conducted using convenience sampling. This may have limited the generalisability of the results. Second, the study was conducted during the latter phase of the COVID-19 pandemic in Saudi Arabia, during which most people had already become accustomed to the virus’ prevalence and the use of protective measures. This may have played an important role in the limited effects on the mental health of the students that were identified. Third, the survey did not include nursing students from private nursing schools. Therefore, their perceptions and attitudes toward the study variables were excluded from this study.

## 5. Conclusions and Recommendations

This study’s results support the conclusion that nursing students in Saudi Arabia experienced few mental health problems during the latter phase of the pandemic, and show that during the pandemic, student nurses had positive attitudes toward the nursing profession. Furthermore, the vast majority decided to continue with nursing despite their experiences during the pandemic. Being female, knowing someone in an immediate social environment who was infected with COVID-19, having high confidence in the government’s response to the pandemic, having low levels of COVID-19-related fear and anxiety, and having high levels of COVID-19 phobia were key factors that were associated with positive attitudes toward nursing. Moreover, living in a rural community, having family members in the nursing profession, having low levels of COVID-19-related anxiety, and having positive attitudes toward nursing increased the odds of nursing students wishing to continue with the nursing profession during the COVID-19 pandemic. This finding suggests that nursing schools should prioritise educational progress, helping students to develop coping strategies, and reducing the psychological effect of the pandemic. This could be done through strategies such as support programs that are student-centred. Furthermore, this study has shown that the COVID-19 pandemic has impacted the psychology of nursing students. Therefore, vulnerable students should be appropriately assessed to aid in the mitigation of the global psychological effect of the pandemic and its related uncertainty.

## Figures and Tables

**Table 1:** Demographic and COVID-19-related perceptions and experiences of the participants (*n* = 726).

Variable	Mean (SD)	Range	*n*	%
Demographic characteristics				
Age	23.24 (4.83)	18–45		
Gender				
Male			235	32.4
Female			491	67.6
Year in the BSN				
Preparatory/year 1			60	8.3
Year 2			253	34.8
Year 3			136	18.7
Year 4			164	22.6
Internship year			113	15.6
Community				
Rural			394	54.3
Urban			332	45.7
Family structure				
Nuclear			437	60.2
Extended			289	39.8
Has a family member/s who is/are nurse				
No			327	45.0
Yes			399	55.0
COVID-19-related perceptions and experiences				
Previously diagnosed with COVID-19				
No			387	53.3
Yes			339	46.7
Family member/s diagnosed with COVID-19				
No			203	28.0
Yes			523	72.0
Know someone in immediate social environment who were infected with COVID-19				
No			180	24.8
Yes			546	75.2
Perceived seriousness of COVID-19 pandemic in Saudi Arabia	47.73 (27.54)	0–100		
Confidence in the pandemic response of the government				
Not at all confident			10	1.4
Not too confident			29	4.0
Somewhat confident			125	17.2
Very confident			562	77.4

**Table 2:** Descriptive analyses of the study variables (*n* = 726).

Variable	Minimum	Maximum	Mean	SD
Fear of COVID-19 Scale	7.00	35.00	12.78	5.90
Coronavirus Anxiety Scale	0.00	15.00	1.17	2.61
Depression, Anxiety, and Stress Scale short form (COVID-19-related stress)	0.00	40.00	7.60	8.22
COVID-19 Phobia Scale	20.00	97.00	38.12	16.64
Obsession with COVID-19 Scale	0.00	16.00	2.48	3.08
Attitude Scale for Nursing Profession				
Preference for the nursing profession	1.00	5.00	4.16	0.74
General position of the nursing profession	1.89	4.78	3.77	0.46
Willingness to continue with nursing as future profession	*n*	%		
Yes	624	86.0		
No	40	5.5		
Not sure	62	8.5		

**Table 3:** Results of the multiple linear regression on the attitudes of students toward the nursing profession (*n* = 726).

Predictor Variables	ß	SE-*b*	Beta	*t*	*p*	95% Confidence Interval	
						Lower	Upper
Age	−0.01	0.01	−0.06	−1.55	0.121	−0.02	0.00
Gender	0.13	0.04	0.11	3.21	0.001 **	0.05	0.22
Year in the BSN	−0.01	0.02	−0.03	−0.82	0.412	−0.05	0.02
Community	0.04	0.04	0.03	0.96	0.340	−0.04	0.11
Family structure	0.04	0.04	0.04	1.11	0.269	−0.03	0.12
Diagnosed with COVID-19 infection	0.02	0.05	0.01	0.35	0.730	−0.07	0.10
Family member/s diagnosed with COVID-19 infection	0.02	0.05	0.02	0.41	0.685	−0.08	0.12
Know someone in immediate social environment who were infected with COVID-19	0.13	0.05	0.10	2.73	0.007 **	0.04	0.22
With family member/s who is/are nurse/s	−0.06	0.04	−0.05	−1.46	0.144	−0.14	0.02
Perceived seriousness of the COVID-19 pandemic in Saudi Arabia	0.00	0.00	0.04	0.99	0.322	0.00	0.01
Confidence in the pandemic response of the government	0.13	0.03	0.14	3.89	<0.001 ***	0.06	0.19
Fear of COVID-19	−0.03	0.01	−027	−4.97	<0.001 ***	−0.04	−0.02
COVID-related anxiety	−0.03	0.01	−0.13	−2.73	0.006 **	−0.05	−0.01
COVID-related stress	0.00	0.00	−0.03	−0.79	0.429	−0.01	0.00
COVID-19 phobia	0.01	0.00	0.16	2.89	0.004 **	0.00	0.01
Obsession with COVID-19	−0.01	0.01	−0.07	−1.60	0.110	−0.03	0.00

Note. The dependent variable was the overall mean score of the nursing students’ attitudes toward the nursing profession. ß is the unstandardised coefficient; SE-b is the Standard error. *R*^2^ = 0.165, Adjusted *R*^2^ = 0.147. ** Significant at 0.01 level, *** Significant at 0.001 level.

**Table 4:** Result of the binary logistic regression on the willingness of the students to continue with nursing as their future profession (*n* = 726).

Predictor Variables	ß	SE	Wald	*p*	Exp(ß)	95% C.I. for Exp(ß)	
						Lower	Upper
Age	−0.05	0.03	2.76	0.097	0.96	0.90	1.01
Gender (Ref.: Female)	0.22	0.29	0.55	0.458	1.24	0.70	2.18
Year in the BSN	−0.20	0.12	3.05	0.081	0.82	0.65	1.03
Community (Ref.: Urban)	0.60	0.28	4.65	0.031 *	1.83	1.06	3.17
Family structure (Ref.: Extended)	0.19	0.28	0.47	0.492	1.21	0.70	2.07
Previously diagnosed with COVID-19 (Ref.: No)	0.13	0.30	0.20	0.654	1.14	0.64	2.06
Family member/s diagnosed with COVID-19 (Ref.: No)	−0.30	0.33	0.82	0.366	0.74	0.39	1.42
Know someone in immediate social environment who were infected with COVID-19 (Ref.: No)	0.40	0.32	1.61	0.205	1.49	0.80	2.78
With family member/s who is/are nurse (Ref.: No)	0.79	0.29	7.39	0.007 **	2.21	1.25	3.92
Perceived seriousness of COVID-19 pandemic in Saudi Arabia	−0.01	0.01	1.39	0.239	0.99	0.99	1.00
Confidence in the pandemic response of the government	0.36	0.19	3.62	0.057	1.44	0.99	2.09
Fear of COVID-19	−0.02	0.03	0.46	0.499	0.98	0.92	1.04
COVID-19-related anxiety	0.08	0.06	1.83	0.176	1.08	0.97	1.22
COVID-19-related stress	−0.04	0.02	4.67	0.031 *	0.96	0.93	1.00
COVID-19 phobia	−0.01	0.01	0.19	0.662	0.99	0.97	1.02
Obsession with COVID-19	0.06	0.06	1.19	0.275	1.06	0.95	1.18
General position of the nursing profession	0.06	0.27	0.05	0.829	1.06	0.625	1.80

Note. * Significant at 0.05, ** Significant at 0.01.

## Data Availability

Data are available upon request.
